# Removal of lipopolysaccharide from protein solution using nanostructured porous supports bearing lipid membranes

**DOI:** 10.1186/1556-276X-8-460

**Published:** 2013-11-05

**Authors:** Masa-aki Wakita

**Affiliations:** 1 Kurita Global Technology Center, Kurita Water Industries, Ltd., 1-1 Kawada, Nogi-machi, Shimotsuga-gun, Tochigi 329-0105, Japan

**Keywords:** Lipid membranes, Lipopolysaccharide, Chitosan

## Abstract

Polymeric lipid membranes of *N*-octadecylchitosan, which consists of 70 mol% of 2-(octadecylamino)-2-deoxy-d-glucopyranose, 17 mol% of 2-amino-2-deoxy-d-glucopyranose, and 13 mol% of 2-acetamido-2-deoxy-d-glucopyranose, were covalently immobilized to carboxylated porous supports composed of chitosan and used for the adsorption of pyrogenic lipopolysaccharide. When human serum albumin solution, including 5 mg mL^-1^ of albumin and 5.6 ng mL^-1^ of lipopolysaccharide, was passed through a column packed with the resulting porous supports bearing lipid membranes assembled in nanoscale, lipopolysaccharide was removed to as low as a detection limit of 0.020 ng mL^-1^ with a quantitative recovery of protein. On the other hand, in the case of directly N-octadecylated porous supports having cationic and hydrophobic ligands which are not assembled as lipid membranes, lipopolysaccharide could not be removed to the detection limit and protein recovery was lower than the porous supports bearing lipid membranes. The difference above as well as difference from conventional adsorbents suggested that the selectivity was attributable to an interaction between the cationic lipid membranes of *N*-octadecylchitosan and lipopolysaccharide as well as protein. The porous supports bearing lipid membranes were stable in 0.5 M NaOH and 0.1 M HCl at ambient temperature. Considering the confirmed excellent selectivity and chemical stability, their practical use as separation media in the pharmaceutical manufacturing can be expected.

## Background

Lipid membranes have been receiving considerable attention as models for various chemical investigations. Densely packed alkyl chains with hydrophilic head groups can have both a hydrophobic interaction and a hydrophilic one with guest substrates. This specific environment provided by lipid membranes is essential for molecular recognition in biological membranes and has been studied in the areas of chemical sensing and separation [1,2].

Proteins or other biologically active substances, especially those produced by recombinant microorganisms, are usually contaminated with lipopolysaccharide (LPS) [3]. LPS, which originates from an outer membrane of Gram-negative bacteria, consists of a polysaccharide and a terminal lipid A moiety. Lipid A is composed of a diglucosamine that is highly substituted with amide- and ester-linked long-chain fatty acids and negatively charged with phosphate groups. For pharmaceutical uses of those active substances, LPS has to be removed to not higher than 0.1 ng mL^-1^ because of its strong pyrogenicity [4]. Intensive studies have been done on the removal of LPS from protein solutions [5]. For example, LPS was selectively removed by ion-exchange chromatography using DEAE-Sepharose CL-6B [6] and affinity chromatography using adsorbents bearing histidine [7], polymyxin B [8], and polycation [9]. However, the removal of LPS is suggested to be extremely difficult when LPS is associated with protein to be purified [5] and has been an issue in pharmaceutical technology and science.

**Figure 1:**
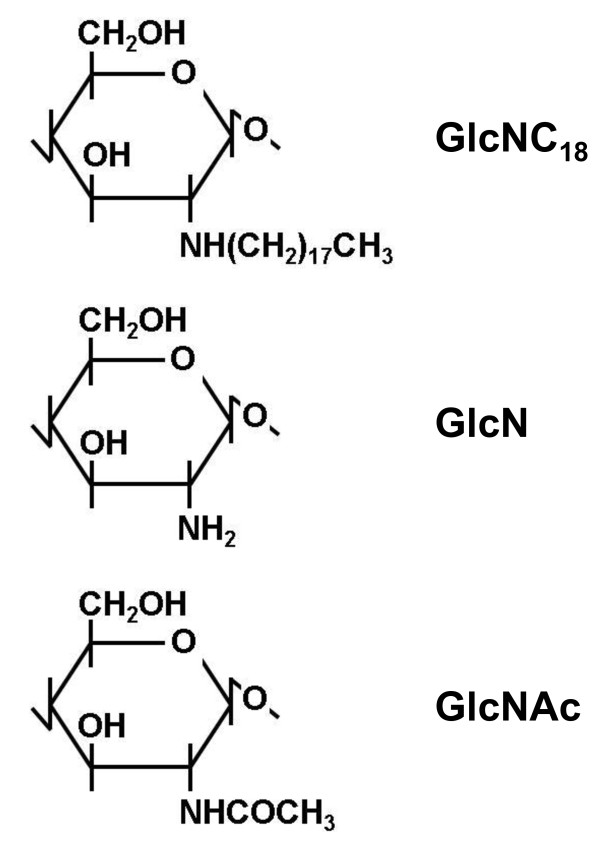
**Monosaccharide components of *N*-octadecylchitosan.**

We have reported the covalent immobilization of polymeric lipid membranes of *N*-octadecylchitosan consisting of 2-deoxy-2-octadecylamino-d-glucopyranose (GlcNC_18_; Figure 1), 2-amino-2-deoxy-d-glucopyranose (GlcN), and 2-acetamido-2-deoxy-d-glucopyranose (GlcNAc) to carboxylated porous supports composed of chitosan [10], and the selective removal of LPS from bovine serum albumin (BSA) solution using the resulting porous materials [11]. In this paper, we would like to report further data of a successful LPS removal to as low as 0.02 ng mL^-1^ from human serum albumin (HSA) solution with a quantitative recovery of protein showing the possibility of their practical use.

## Methods

### Materials and general methods

Chitosan was purchased from Dai-ichi Kogyo Seiyaku Co., Ltd. (Kyoto, Japan). The degree of deacetylation was determined as 87 mol% by colloidal titration. Intrinsic viscosity was 1.42 dL g^-1^ (0.2 M CH_3_COOH/0.1 M CH_3_COONa, 30°C) which corresponded to 2.67 × 10^4^ of molecular weight relative to poly(ethylene glycol). A cross-linked porous chitosan having a particle size of 45 to 420 μm and an average pore diameter of 2 μm, a product of Kurita Water Industries, Ltd. (Tokyo, Japan), was used as obtained. 1-Bromooctadecane, succinic anhydride (Kishida Chemical Co., Ltd., Osaka, Japan), 1-ethyl-3-(3-(dimethylamino)propyl)-carbodiimide hydrochloride (water-soluble carbodiimide (WSC), Dojindo Laboratories, Kumamoto, Japan), *N*-hydroxysuccinimide (HOSu), and d-(+)-glucosamine hydrochloride (Tokyo Kasei Kogyo Co., Ltd., Tokyo, Japan) were used as obtained. A volume of adsorbent was determined after standing for 24 h in a measuring cylinder with water. Infrared (IR) spectra were recorded using IR-810 (Jasco Co., Ltd., Tokyo, Japan). Total organic carbon content analysis and differential scanning calorimetry (DSC) were carried out using TOC-5000A (Shimadzu MFG., Kyoto, Japan) and DSC220C (Seiko Instruments Inc., Tokyo, Japan), respectively. Scanning electron micrographs (SEM) and transmission electron micrographs (TEM) were taken at JEOL DATUM (Tokyo, Japan) using JSM-6400 F (JEOL) and JEM-1200EX (JEOL), respectively. DEAE-Sepharose CL-6B and Pyrosep (histidine-immobilized agarose, Sigma-Aldrich, Tokyo, Japan) were obtained from manufacturers. HSA (20% *w*/*v*) and LPS (*Escherichia coli* serotype O127:B8) were products of Nihon Pharmaceutical Co., Ltd. (Tokyo, Japan) and Difco Laboratories (Detroit, MI, USA), respectively, and used as obtained. Toxicolor (Seikagaku Corporation, Tokyo, Japan), which is a chromogenic *Limulus* amebocyte lysate test, was used as an assay method for LPS. Samples containing LPS were diluted with Tris–HCl buffer (pH 8.0) to lower than 0.085 ng mL^-1^ of LPS and assayed by the method recommended by the manufacturer. The detection limit of LPS in this test was as low as 0.020 ng mL^-1^, which corresponded to 0.06 endotoxin unit. HSA concentration was measured by UV at 236 nm to avoid interference of a stabilizer *N*-acetyltryptophan showing adsorption at 280 nm.

### Preparation of porous supports bearing lipid membranes

**Figure 2:**
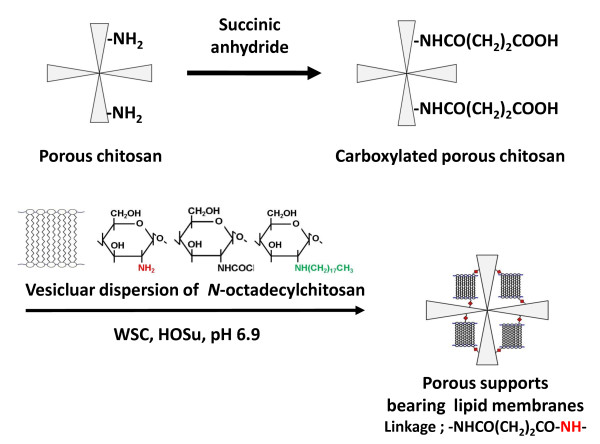
Preparation schemes of the porous supports bearing lipid membranes.

Preparation of porous supports bearing lipid membranes is described briefly with the conceptual scheme (Figure 2). Chitosan was simply N-alkylated by 1-bromooctadecane in *N*,*N*-dimethylacetamide to yield *N*-octadecylchitosan consisting 70 mol% of GlcNC_18_, 17 mol% of GlcN, and 13 mol% of GlcNAc. In DSC of *N*-octadecylchitosan, an endothermic peak was observed (*T*_*c*_ = 46°C) indicating a gel to liquid-crystalline phase transition. Dispersion liquid was prepared by suspending *N*-octadecylchitosan in water including hydrochloric acid and successive sonication. Electron microscopic observation of the dispersion liquid revealed the existence of unilamellar vesicles having diameters of 10 to 150 nm [12]. Carboxylated porous supports were prepared by N-succinylation of the cross-linked porous chitosan with succinic anhydride. Vesicular dispersion of *N*-octadecylchitosan was reacted with the carboxylated porous supports in the presence of WSC and HOSu to form amide bonds from primary amino groups of *N*-octadecylchitosan and carboxyl groups of the porous supports. The resulting materials were further reacted with *N*-acetylglucosamine to block the remaining carboxyl groups by amidation [10].

### Direct alkylation of porous supports

After being washed by dimethylacetamide, the cross-linked porous chitosan (20.0 mL, including 4.6 mmol of NH_2_) was reacted with mild stirring using 1-bromooctadecane (4.56 g, 13.7 mmol) in the presence of Na_2_CO_3_ (1.45 g, 13.7 mmol) at 70°C for 69 h in dimethylacetamide under nitrogen atmosphere. Particles were recovered by filtration and washed with water, warm ethyl acetate, ethanol, and water successively. Changes in the appearance and porous structure were not observed by SEM. In the IR spectra (KBr pellet), *ν*_CH_ of CH_2_ 2,925 and 2,850 cm^-1^ was observed. Ion-exchange capacity was 2.3 meq g^-1^ dry particles. Using this value, the colloidal equivalent of chitosan (5.0 meq g^-1^, pH 4.0) used for the preparation of the cross-linked porous chitosan and the ion-exchange capacity of the cross-linked porous chitosan (3.8 meq g^-1^) which was cross-linked by 1,6-diisocyanatohexane, GlcN of the cross-linked porous chitosan, and GlcNC_18_ of the resulting directly alkylated porous supports were calculated as 69 and 47 mol%, respectively. Therefore, about half of the monosaccharide units of the support particles are deemed to be octadecylated.

### Column-wise adsorption of LPS from protein solutions

Purified water and buffer solutions were sterilized using an autoclave at 115°C to 121°C for 15 min. Glass wares were also sterilized using the autoclave at 250°C for 2 h. LPS aqueous solution, which was prepared by vortex mixing and dilution with water, was added to HSA preparation. The solution was filtered with a filter disk having 0.2-μm-diameter pores and diluted with a buffer solution to a desired concentration for column-wise experiments. Phosphate buffer was used for experiments at pH 7.0 and 8.0, and acetic acid buffer was used for those at pH 4.3 and 5.3. Buffer solutions were prepared by adding NaOH solution of a predetermined concentration to phosphoric acid or acetic acid to obtain the desired ionic strength (*μ*).

Column-wise adsorption was done at 20°C. Adsorbents were suspended in water and fed into a glass column (8 mm i.d. × 100 mm length) using a LC-6A pump (Shimadzu Corp., Kyoto, Japan). The length of the gel bed was between 930 and 980 mm. The resulting column was washed with 0.5 M NaOH, at 10 mL h^-1^ for 3 h, and left overnight filled with 0.5 M NaOH to decompose LPS in the column (depyrogenation). After washing with water for 3 h, 0.1 M acetic acid was passed through for 1.5 h to convert amino groups of *N*-octadecylchitosan immobilized on the supports to their ammonium forms. After the buffer solution was passed through for 6.5 h, HSA solution was passed through at 5 mL h^-1^ for 15 to 16 h. The eluted solution was collected immediately as ten fractions of 7.5 mL each. Fractions of 2, 4, 6, 8, and 10 were analyzed for the concentrations of LPS and HSA.

### Chemical stability

Porous supports bearing lipid membranes of *N*-octadecylchitosan were immersed in 0.5 M NaOH or 0.1 M HCl at ambient temperature overnight and then washed with water and subjected to IR spectroscopic analysis. The supports (20 mL) were packed into a column (XK16/20, Pharmacia LKB Biotechnology, Uppsala, Sweden; 16 mm i.d. × 100 mm length). The packed column was filled with 0.5 M NaOH and allowed to stand overnight at 20°C. After washing with 200 mL of water, 50 mL of water was circulated in the column for 24 h at a flow rate of 1 BV h^-1^. The water was recovered and subjected to an analysis of total organic carbon content [10].

## Results and discussion

### Porous supports bearing lipid membranes

**Figure 3:**
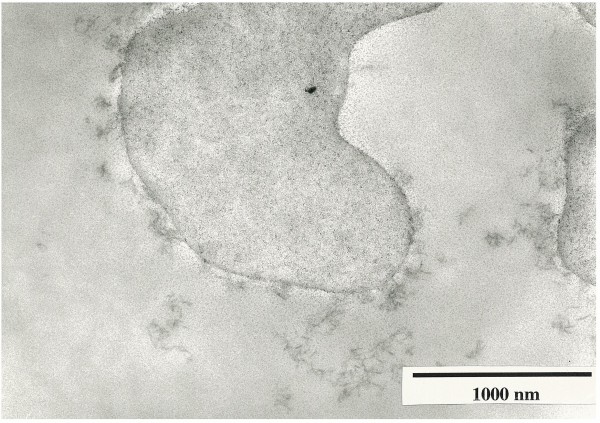
**Transmission electron micrograph of the porous supports bearing lipid membranes (ultrathin section).** ×60,000 as provided.

Characterization of the porous supports bearing lipid membranes was reported previously [10]. An IR spectrum of the cross-linked porous chitosan reacted with succinic anhydride showed a new absorption band at 1,720 cm^-1^ (ν_C=O_ of COOH) and an increase of intensity at 1,655 and 1,560 cm^-1^ (ν_C=O_ of NHCO) indicating selective N-succinylation. After further reaction with the vesicular dispersion of *N*-octadecylchitosan, a small but distinct increase of ν_CH_ at 2,925 cm^-1^ and a disappearance of ν_C=O_ of COOH at 1,720 cm^-1^ were observed. The difference spectrum, *N*-octadecylchitosan-immobilized supports minus carboxylated ones, demonstrated ν_CH_ of *N*-octadecylchitosan methylenes at 2,925 and 2,850 cm^-1^ and ν_C=O_ of NHCO at 1,655 and 1,560 cm^-1^. These results supported the covalent immobilization of *N*-octadecylchitosan to the carboxylated supports by amide bonds. A rougher surface was observed at the scanning electron micrograph of *N*-octadecylchitosan-immobilized supports compared to carboxylated ones. Furthermore, threadlike materials in order of tens of angstrom thickness were observed around the fibrous support in TEM of ultrathin sections of the *N*-octadecylchitosan-immobilized supports (Figure 3). From the above results, polymeric lipid membranes of *N*-octadecylchitosan were covalently immobilized to porous supports. The immobilized amount of *N*-octadecylchitosan was estimated as 4 mg mL^-1^ of particles from the consumption of hydrochloric acid in titration.

### Column-wise adsorption of LPS from protein solution by porous supports bearing lipid membranes

**Figure 4:**
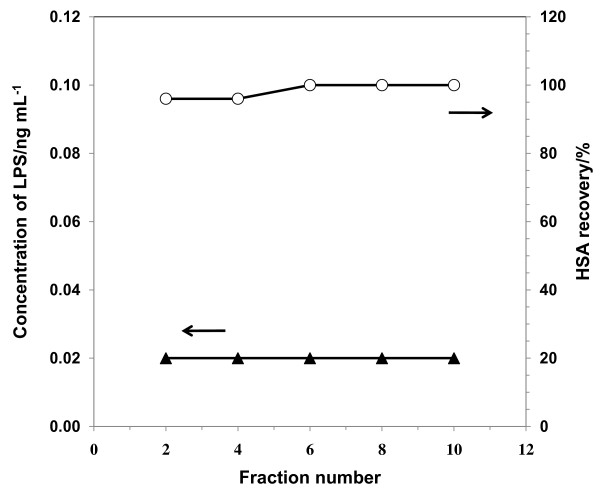
**Elution profile of LPS and HSA from the column packed with porous supports bearing lipid membranes.** HSA, 5 mg mL^-1^; LPS, 5.6 ng mL^-1^; pH, 7.0; ionic strength, 0.1. Since concentrations of LPS in all fractions were lower than the detection limit, they were plotted at the detection limit of 0.02 ng mL^-1^. Concentration of LPS (filled triangle) and recovery of HSA (open circle).

For the porous supports bearing lipid membranes, it was reported that LPS was removed to as low as 0.1 ng mL^-1^ from the BSA solution at pH 4.3 to 7.0 with the ionic strength of 0.01 to 0.1 with a quantitative recovery of protein [11]. BSA was highly contaminated by LPS as obtained with the concentration of 100 to 148 ng mL^-1^ of LPS for 5 mg mL^-1^ of BSA. In this report, the column-wise adsorption experiments using HSA were carried out for not only the porous supports bearing lipid membranes but also the conventional adsorbents for LPS removal. The HSA/LPS mixed solution was passed through the column packed with the adsorbents. Concentrations of HSA and LPS were 5 mg mL^-1^ and 1 to 39 ng mL^-1^, respectively. The HSA used in this paper was produced from donated blood, and the LPS concentration in the original HSA solution was lower than the detection limit. Since concentrations of LPS and recoveries of HSA of recovered fractions were relatively constant as shown in Figure 4 of an elution profile example, the results of the column-wise adsorption were summarized by an average value of fractions in Tables 1 and 2.

**Table 1:** Column-wise adsorption of LPS and HSA using the porous supports bearing lipid membranes

**Run**	**Solution applied**^**a**^			**Solution recovered**^**b**^		
	**pH**	**Ionic strength**	**LPS**	**LPS**	**HSA**	
			**Concentration (ng mL**^**-1**^**)**	**Concentration (ng mL**^**-1**^**)**	**Removal (%)**	**Recovery (%)**
1	4.3	0.01	4.2	0.039	99.1	101
2	5.3	0.1	3.6	<0.020	99.4<	100
3	7.0	0.1	5.6	<0.020	99.6<	100
4	8.0	0.05	3.2	<0.020	99.4<	100

^a^The concentration of HSA is 5 mg mL^-1^; ^b^LPS concentration, LPS removal, and HSA recovery are averages of recovered fractions. The adsorption capacity of the porous supports bearing lipid membranes was estimated as >2.36 × 10^4^ EU mL^-1^ adsorbent by other runs at pH 4.3, *μ* = 0.05.

**Table 2:** Column-wise adsorption of LPS and HSA using various adsorbents

**Run**	**Adsorbent used**	**Solution applied**^**a**^	**Solution recovered**^**b**^		
		**LPS**	**LPS**		**HSA**
		**Concentration (ng mL**^**-1**^**)**	**Concentration (ng mL**^**-1**^**)**	**Removal (%)**	**Recovery (%)**
3	Porous supports bearing lipid membranes	5.6	<0.020	99.6	100
5	DEAE-Sepharose CL-6B	39	0.079	99.8	37
6	Pyrosep; histidine-immobilized agarose	38	0.110	99.7	104
7	Directly alkylated porous chitosan	3.2	0.058	98.2	96

^a^HSA concentration, 5 mg mL^-1^; pH, 7.0; ionic strength, 0.1; ^b^LPS concentration, LPS removal, and HSA recovery are averages of recovered fractions.

As shown in Table 1, in the case of the porous supports bearing lipid membranes, LPS was removed to lower than 0.020 ng mL^-1^ at pH 5.3, 7.0, and 8.0 and to 0.039 ng mL^-1^ at pH 4.3 with a quantitative recovery of protein. In the case of DEAE-Sepharose CL-6B and histidine-immobilized agarose (Table 2), concentrations of LPS in the recovered solution were higher than those in the porous supports bearing lipid membranes. Since the removal of LPS to lower than the detection limit is usually required for pharmaceutical applications, the above removal ability of the porous supports bearing lipid membranes can be an advantage in practical use.

### Mechanism of the selective adsorption of LPS

For the argument of adsorption mechanism, the electric charge of LPS and protein, aggregation behavior of LPS, and interaction between LPS and protein should be reviewed. Since lipid A is partially phosphorylated, LPS exhibits a net negative charge at all pH ranges applied. On the other hand, since p*I* of albumin is 4.9, it exhibits a net positive charge at pH 4.3 and a net negative charge at pH 5.3, 7.0, and 8.0. About the aggregation of LPS and the interaction between LPS and proteins, it is well known that LPS forms various molecular aggregates in aqueous solutions [13] and interacts with various proteins to form molecular complexes [5]. From the amphiphilic structure of LPS and the effect of nonionic detergent on the dissociation of LPS [14], the aggregation between LPS is likely caused by hydrophobic interaction between LPS molecules. Considering our dynamic light scattering study showing that LPS interacts with bovine serum albumin [15], it seems that LPS interacts with HSA in applied conditions.

**Figure 5:**
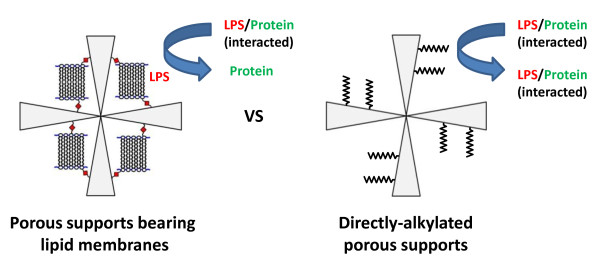
Conceptual diagrams for removal of LPS from protein solution by porous supports bearing lipid membranes.

Based on above information, the removal of LPS to a lower concentration by the porous supports bearing lipid membranes can be attributable to both an electrostatic interaction and hydrophobic one between the cationic lipid membranes of *N*-octadecylchitosan and LPS. The large pore diameter of the support material is also advantageous to incorporate LPS aggregates compared to conventional adsorbents used. The reason why negatively charged HSA is not adsorbed to the cationic porous supports bearing lipid membranes seems to be their low p*Ka*. In our preliminary evaluation, they exhibited p*Ka* of 6 to 9 for primary and secondary amino groups (-NH_2_ and -NHR-) consisting of chitosan and *N*-octadecylchitosan. These values are considerably lower than that of the diethylaminoethyl (DEAE) group (p*Ka*, 11.5) used for usual anion-exchange chromatography and lead to a weak anion-exchange property. The difficulty of hydrophobic adsorption of albumin to lipid membranes in rigid gel phase also seems to be preferable for a good recovery of HSA [15]. It is of interest to confirm if the lipid membrane structure is essential for the LPS removal and protein recovery shown in Table 1. With this consideration in mind, the direct alkylation of the cross-linked porous chitosan was carried out. Although the resulting directly alkylated porous chitosan has a similar surface chemical structure, its alkyl chains are not assembled as lipid membranes. As shown in Table 2, in the case of the directly alkylated porous chitosan, LPS was removed to 0.058 ng mL^-1^ with 96% of HSA recovery. It seems that LPS molecules which interacted with protein could be removed by the porous supports bearing lipid membranes by a strong interaction between LPS and cationic lipid membranes. The structural similarity between LPS and *N*-octadecylchitosan lipid membrane seemed to enhance the interaction too [16]. On the other hand, some of them could not be removed by the directly alkylated one because of a weaker interaction with LPS (Figure 5). Lower HSA recovery by the directly alkylated porous chitosan seems to be caused by a hydrophobic interaction between octadecyl groups and HSA which binds fatty acids.

Specific interaction for LPS of the porous supports bearing lipid membranes seems to be useful in a forthcoming issue. Antibody drugs with superior efficacy have been developed intensively in the last few decades. However, since they are produced by mammalian cells such as Chinese hamster ovary, their cost is very expensive and needs to be reduced. For this issue, intensive researches have been continued for production of antibody drugs using microorganisms such as *E. coli*[17]. Although protein A chromatography is useful to recover antibody drugs from preparations, further chromatography is necessary to obtain the required purity for their clinical use. It is likely that production of antibody drugs by *E. coli* leads to a further requirement of selective removal of LPS. Considering the selectivity for LPS of the porous supports bearing lipid membranes, their application for purification of antibody drugs is interesting.

### Chemical stability of porous supports bearing lipid membranes

The elution property of separation medium is a key issue in liquid purification for the pharmaceutical industry. Elution from porous supports bearing lipid membranes of *N*-octadecylchitosan was evaluated by measuring the total organic carbon content in an eluent from a column packed with 20 mL of supports. The total organic carbon in the recovered water described in the experimental section was 400 μg L^-1^[10]. Even assuming that all of the organic carbons are due to eluted *N*-octadecylchitosan, the eluted *N*-octadecylchitosan (C, 65.7%) is calculated as 3.0 × 10^-5^ g. This amount is 0.038% of the *N*-octadecylchitosan immobilized on the 20-mL supports. This stability for alkali is attributable to the stability of the amide linkage used for the immobilization of *N*-octadecylchitosan as well as the stability of the support material. Any substantial change was not observed in the IR spectra of the porous supports bearing lipid membranes after immersion in 0.5 M NaOH or 0.1 M HCl overnight at ambient temperature. This chemical stability, especially to 0.5 M NaOH immersion which is used as a standard depyrogenation procedure, is robust enough for a practical application in the pharmaceutical industry.

## Conclusions

Porous supports bearing cationic lipid membranes of *N*-octadecylchitosan assembled in nanoscale adsorb LPS selectively from HSA solution at pH 4.3 to 8.0 with the ionic strength of 0.05 to 0.1. LPS was removed to as low as a detection limit of 0.020 ng mL^-1^ by a column-wise adsorption with a quantitative recovery of HSA. Since LPS includes a terminal diglucosamine which is negatively charged and highly substituted with long-chain fatty acids, LPS is adsorbed by both an ionic interaction and a hydrophobic one. In addition, the low p*Ka* of the chitosan-based material as well as the rigid gel phase of lipid membranes leads to a relatively weak interaction between HSA and results in the selective adsorption of LPS. For a practical use in separation, especially such as liquid purification for the pharmaceutical industry, a lower elution property of the separation medium is a key issue. Considering the excellent selectivity and the chemical stability of the supports bearing cationic lipid membranes of *N*-octadecylchitosan, their practical use as separation media in pharmaceutical manufacturing can be expected.

## Competing interests

The author declares that he has no competing interests.
